# Data of an Iranian Population of *L. proximus* Sturhan & Argo, 1983, with taxonomic revision of *L. israelensis* Peneva, Orion, Shlevin, Bar-Eyal & Brown, 1998 (Nematoda: Longidoridae) and Proposal for a New Synonymy

**DOI:** 10.21307/jofnem-2019-054

**Published:** 2019-09-17

**Authors:** Mazdosht Giti, Leila Kashi, Majid Pedram

**Affiliations:** 1 Agricultural Research Center of Hamadan, Hamadan, Iran; 2 Department of Plant Protection, Bu-Ali Sina University, Hamadan, Iran; 3 Department of Plant Pathology, Faculty of Agriculture, Tarbiat Modares University, Tehran, Iran

**Keywords:** ITS, LSU rDNA D2-D3, phylogenetics, taxonomy

## Abstract

The morphology and morphometric characteristics of a *Longidorus* population recovered from a wheat-potato field in Hamadan province, western Iran, fit well with those given for two species, *L. proximus* and *L. israelensis*. The Iranian population was characterized by 5.6 to 8.6-mm-long females having a 17 to 21-µm-wide lip region separated from the rest of the body by a shallow depression, pocket-shaped amphidial fovea with a simple base and a ventral enlargement, a guiding ring at 31 to 40 µm distance from the anterior end, 108 to 127-µm-long odontostyle, 58 to 64-µm-long odontophore, 101 to 129-µm-long pharyngeal bulb with remarkably larger dorsal gland nucleus (at 49 to 53% of the bulb length) and two smaller ventrosublateral nuclei (at 66 to 76% of the pharyngeal bulb length), four juvenile developmental stages, and a rare male. The morphological and molecular data corroborated its assignment to the species *L. proximus*. In molecular phylogenetic analyses using partial LSU rDNA D2-D3 sequences, the presently studied Iranian population and previously sequenced isolates of *L. proximus* formed a clade with *L. cretensis*, *L. iranicus*, *L. pseudoelongatus*, and *L. closelongatus*, all except *L. pseudoelongatus* with no available data, having the similar pharyngeal gland nuclei size and arrangement. In internal transcribed spacer 1 (ITS1) phylogeny, it formed a clade with *L. sturhani* and four aforementioned species. The characters delimiting the two species *L. proximus* and *L. israelensis* were discussed and a new synonymy was proposed.

*Longidorus proximus* (Sturhan and Argo, 1983) was originally described as a parthenogenetic species. Later, a bisexual population was reported by Roca (1986). The species was reported from Iran in a conference abstract, but the morphological and morphometric data of this population were not available (Niknam et al., 2006).

*Longidorus israelensis* (Peneva et al., 1998) is currently only known by its type population (Peneva et al., 1998) and has not been reported since its description. It was described on the basis of its morphological characteristics; the information on juvenile developmental stages, a tentative male, and molecular data were lacking. Recent studies (Zhao et al., 2017), however, emphasize using molecular data for reliable identification of cryptic species, especially for economically important and quarantine pests. The history of the reported *Longidorus* (Mikoletzky, 1922) species in Iran is given by Gharibzadeh et al. (2018). Some recent studies in Iran have focused on the molecular taxonomy of longidorids in Iran (Jahanshahi Afshar, 2019; Jahanshahi Afshar et al., 2019; Mirzaie Fouladvand et al., 2019; Mobasseri et al., 2019). During the present study, a population of *Longidorus* was recovered from a wheat-potato field in Hamadan province and was studied using morphological and molecular criteria. The recovered population looked similar to two species, *L. proximus* and *L. israelensis*, mainly by the shape of amphidial fovea and characteristics of the pharyngeal bulb, i.e., the arrangement and size of the glands nuclei. Thus, the present study aims to identify the recently recovered population of *Longidorus* and discuss on the taxonomy of *L. israelensis*.

## Materials and methods

### Sampling, nematode extraction, mounting, and morphological studies

A total number of 35 soil samples were collected from wheat and potato fields in the city of Hamadan during a survey to identify longidorid nematodes occurring in these fields. The soil samples were collected from 20 to 40 cm depth in May 2016. The longidorid nematodes were extracted by suspending the soil samples in water and collecting the specimens using 20 and 60-mesh (US standard mesh numbers, equal to 841 and 250-μm openings) sieves. The specimens studied here were recovered from a field with a wheat-potato rotation culture, hand-picked using a Nikon SMZ1000 stereomicroscope, heat-killed by adding boiling 4% formaldehyde solution, and transferred to anhydrous glycerin according to De Grisse (1969). Measurements were made using a drawing tube attached to an Olympus BX-41 light microscope. The juvenile stages were identified according to Robbins et al. (1995). The digital images were prepared using an Olympus DP72 digital camera attached to an Olympus BX51 microscope powered with differential interference contrast (DIC).

### DNA extraction, PCR, and sequencing

For the molecular phylogenetic studies, two live nematode specimens were picked out, studied individually on temporary slides, photographed, and transferred to a small drop of TE buffer (10 mM Tris-Cl, 0.5 mM EDTA; pH 9.0, QIAGEN Inc., Valencia, CA) individually on separate clean slides, and each specimen was squashed using a clean slide cover glass. The suspension was collected by adding 50 μl TE buffer. Each sample was regarded as an independent DNA sample, and stored at −20°C until used as polymerase chain reaction (PCR) template. Primers used for the PCR amplification of the D2–D3 expansion domains of the LSU rDNA were forward D2A (5′-ACAAGTACCGTGAGGGAAAGTTG-3′) and reverse D3B (5′-TCGGAAGGAACCAGCTACTA-3′) (Nunn, 1992) primers. The internal transcribed spacer 1 (ITS1) fragment was amplified using the forward primer rDNA1 (5′-TTGATTACGTCCCTGCCCTTT-3′) and the reverse primer rDNA1.58s (5′-ACGAGCCGAGTGATCCACCG-3′) (Subbotin et al., 2000). PCR was carried out for both the aforementioned fragments in a total volume of 40 μl (12 μl distilled water, 20 μl 2x Master mix (Ampliqon, Denmark), 2 μl of each primer (10 pMol/μl), and 4 μl of DNA template). The thermal cycling program for both reactions was as follows: denaturation at 94°C for 5 min, followed by 32 cycles of denaturation at 94°C for 30 sec, annealing at 52°C for 40 sec, and extension at 72°C for 80 sec. A final extension was performed at 72°C for 10 min. The PCR products were purified and sequenced directly for both strands using the same primers with an ABI 3730XL sequencer (Bioneer Corporation, South Korea). The newly obtained sequences were submitted to the GenBank database under the accession numbers given in LSU and ITS1 trees.

### Phylogenetic analyses

The newly generated sequences were compared with the available sequences in the GenBank database using the basic local alignment search tool (BLAST) (https:// blast.ncbi.nlm.nih.gov/Blast.cgi). For LSU phylogeny, several available sequences of the genus were retrieved from the database (a large and a smaller pruned LSU datasets were prepared). The currently available ITS1 sequences of *Longidorus* spp. were retrieved for the ITS1 phylogeny. The LSU sequences were aligned using ClustalX2 (www.clustal.org). The ITS1 dataset was aligned using MUSCLE as implemented in MEGA (Tamura et al., 2013), and the alignment of both datasets was edited using MEGA (Tamura et al., 2011). The appropriate model of base substitution was selected using MrModeltest 2 (Nylander, 2004). The Akaike-supported model, a general time-reversible model, including among-site rate heterogeneity and estimates of invariant sites (GTR+G+I), was selected and used in the phylogenetic analyses of both LSU and ITS1 datasets. Bayesian analyses were performed with MrBayes 3.1.2 (Ronquist and Huelsenbeck, 2003) by running the chains for five million generations for the three aforementioned analyses (the large and pruned LSU datasets, and the ITS1 dataset). After discarding burn-in samples and evaluating convergence, the remaining samples were retained for further analyses. The Markov chain Monte Carlo method within a Bayesian framework was used to estimate the posterior probabilities of the phylogenetic trees (Larget and Simon, 1999) using the 50% majority rule. For maximum likelihood (ML) analyses, raxmlGUI version 1.1 (Silvestro and Michalak, 2012) was used and the analyses were performed using the same model of nucleotide substitution in Bayesian inference (BI) (GTR+G+I) for the pruned LSU and ITS1 trees. For phylogenetic analyses of LSU dataset, *Nevadanema nevadense* (Álvarez-Ortega and Pena-Sañtiago, 2012) (JN242245) and *Prodorylaimus* sp. (EF207241) were used as outgroup taxa. *Xiphinema index* (Thorne and Allen, 1950) (HG969306) and *X. vuittenezi* (Luc et al., 1964) (HG969309) were used as the outgroup taxa in ITS1 tree. The output files of the used phylogenetic programs were visualized using Dendroscope V.3.2.8 (Huson and Scornavacca, 2012) and redrawn using CorelDRAW software version 13. The Bayesian posterior probability (BPP) and ML bootstrap (BS) values exceeding 50% are given on appropriate clades in the shape of BPP/ML BS.

## Results and description

### Iranian population of *Longidorus proximus*

***= L. israelensis*** **syn. n.**

#### Measurements

See Table 1.

**Table 1.** Morphometrics of Iranian population of *Longidorus proximus* (Sturhan & Argo, 1983).

Stage/character	J1	J2	J3	J4	Female	Male
n	1	3	12	4	19	1
L	1,792	2,197.3 ± 78.0 (2,125–2,280)	3,141.7 ± 413.6 (2,540–3,892)	4,762.8 ± 537.6 (4,395–5,547)	6,728.4 ± 654.2 (5,600–8,570)	9,167
a	69	73.2 ± 0.3 (72.9–73.5)	85.9 ± 5.2 (75.8–93.7)	109 ± 5 (101.7–112.7)	111.6 ± 11.4 (96–142)	143.2
b	72	6.7 ± 0.5 (6.1–7.1)	9.2 ± 1.5 (7.5–12.1)	10.9 ± 0.9 (10.2–12.2)	13.8 ± 1.5 (11.6–17.0)	203.7
c	37	50.8 ± 6.1 (43.7–54.5)	71.7 ± 6.8 (62–84)	114.3 ± 15.0 (102.2–135.3)	166.2 ± 15.6 (141.8–191.0)	191
c'	3	2.1 ± 0.2 (2.0–2.3)	1.6 ± 0.1 (1.4–1.7)	1.2 ± 0.1 (1.1–1.3)	0.9 ± 0.1 (0.7–1.1)	0.7
V	–	–	–	–	53.8 ± 2.9 (49.7–61.2)	–
Odontostyle	67	74.0 ± 1.5 (72–76)	78.8 ± 1.0 (82–84)	100 ± 4 (96–104)	117.5 ± 6.5 (108–127)	119
Replacement odontostyle	77	94.5 ± 6.5 (89–101)	94.5 ± 10.0 (92–111)	113 ± 6 (105–118)	–	–
Odontophore	24	38 ± 5 (33–44)	36.5 ± 3.5 (33–40)	47.5 ± 7.5 (36–52)	60.5 ± 2.5 (58–64)	66
Total stylet	91	112.5 ± 5.5 (108–119)	119.5 ± 2.5 (117–122)	146.5 ± 9.0 (135–156)	178.0 ± 7.5 (169–189)	175
Width of lip region	10	10.8 ± 1.6 (9–12)	13.7 ± 0.7 (12.5–15.0)	16.3 ± 1.0 (15–17)	18.7 ± 1.0 (17–21)	19
Pharynx	250	329.7 ± 28.6 (300–357)	345.0 ± 29.7 (285–380)	435 ± 28 (400–460)	477 ± 21.3 (443–512)	480
Body width at mid-body	26	30 ± 1 (29–31)	36.7 ± 5.2 (30–47)	43.8 ± 5.2 (39–50)	61 ± 7 (50–75)	64
- at base of pharynx	25	28 ± 0 (28–28)	33.6 ± 3.1 (29–38)	39.6 ± 2.1 (37–42)	50.4 ± 4.4 (45–63)	48
- at anus level	20	21.2 ± 1.0 (20–22)	27.2 ± 2.4 (23–31)	35.0 ± 1.8 (33–37)	45.2 ± 3.8 (41–55)	45
- at guiding ring level	15	16.7 ± 0.6 (16–17)	19.9 ± 0.8 (19.0–21.5)	23.1 ± 0.9 (22–24)	27.1 ± 1.6 (25–32)	28
Anterior end to guiding ring	18	22.7 ± 1.5 (21–24 )	25.8 ± 2.1 (23–29)	29.3 ± 1.0 (28–30)	35.4 ± 2.7 (31–40)	36
- to vulva	–	–	–	–	3,619 ± 356 (2,900–4,590)	–
Tail length	49	43.7 ± 5.7 (39–50)	43.8 ± 3.7 (39–50)	41.8 ± 1.0 (41–43)	41.0 ± 3.3 (35–46)	48

Note: Measurements are in µm and all data are in the form mean ± S.D. (range).

Figures 1 and 2.

**Figure 1:**
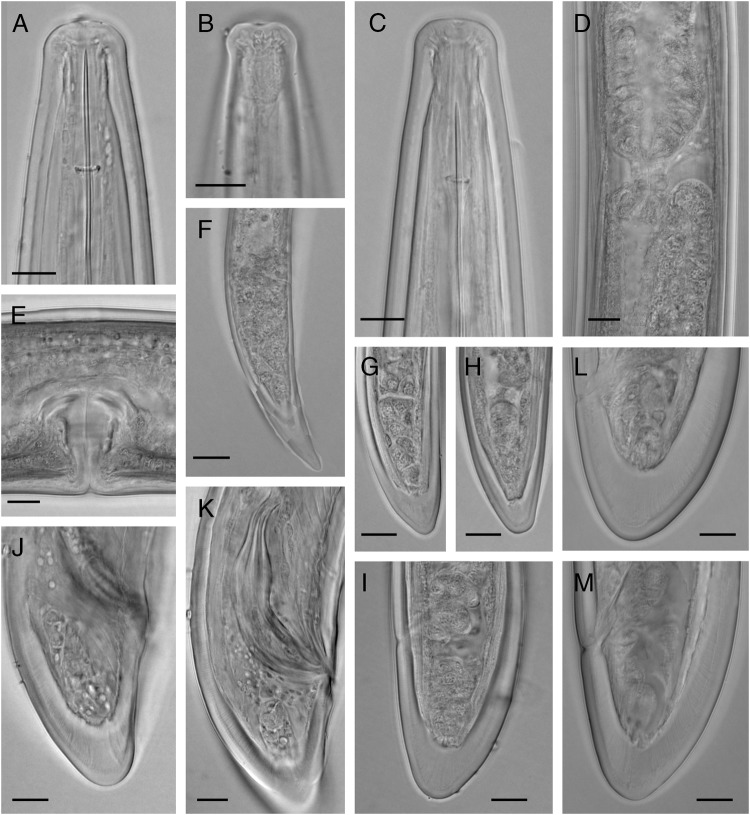
Light micrographs of Iranian population of *Longidorus proximus* (Sturhan & Argo, 1983). (A–C) Anterior region (B: Amphidial pouch), (D) Junction of uterus and *pars dilatata oviductus* (sphincter), (E) Vagina, (F–I) Tail of J1–J4, respectively, (J&K) Male tail, L&M: Female tail. (scale bars = 10 µm).

**Figure 2:**
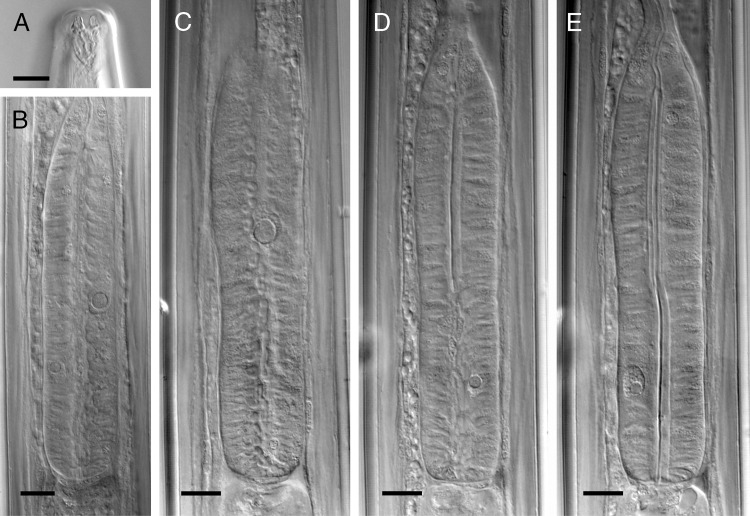
Light micrographs of *Longidorus proximus* (Sturhan & Argo, 1983). (A) Amphidial pouch, (B) The dorsal gland nucleus and one of ventrosublateral nuclei in female code Ham1, (C–E) Pharyngeal glands nuclei in female code Ham2 (C: Dorsal gland’s nucleus, (D,E) ventrosublateral glands nuclei). (scale bars = 10 µm).

### Material examined

19 females, one male and 20 juveniles collected from a wheat/potato field in the city of Hamadan, Hamadan province, western Iran. GPS coordinates: N 34˚54′12.63″, E 48˚31′03.84″.

#### Females

Body slender, gradually tapering toward the anterior end, curved into C-shape on relaxation. Cuticle 3.5 to 5.5 µm thick in the anterior region at level with the guiding ring, 3.7 to 4.6 µm thick at the mid-body, 4.5 to 7.0 µm thick at the anus, and 15 to 21 µm thick at the tail end. Lip region expanded, rounded at the corners, separated from the rest of the body by a shallow depression. Amphidial fovea pocket-shaped, their base simple with a ventral enlargement. Odontostyle 1.7 to 2.0 times the odontophore length. Guiding ring at 1.5 to 1.9 times lip region width from the anterior body end. Nerve ring slightly posterior to the base of odontophore in resting position of the stylet. Pharynx dorylaimoid, anterior slender part flexible, posteriorly expanding to a muscular terminal bulb 4.8 to 5.6 times longer than wide, with three nuclei. Remarkably large dorsal gland nucleus (DN), with 4 to 5 µm diameter, at 49 to 53% of the bulb length, the two ventrosublateral nuclei (S1N) smaller, with 1.8 to 3.3 µm diameter, both at about the same level and 66 to 76% of the pharyngeal bulb length (Fig. 2) (Loof and Coomans, 1972). Cardia slender, 13 to 23 μm high, 8 to 13 μm wide. Prerectum 510 to 755 μm long or 13 to 24 times, and rectum 17 to 47 μm long or 0.6 to 2.3 times the anal body width. Reproductive system amphidelphic, with both branches almost equally developed, anterior branch 462 to 880 μm long, posterior branch 357 to 867 μm long, each composed of a reflexed ovary, oviductus with a well-developed *pars dilatata oviductus*, tubular uterus, vagina 31 to 44 µm or 50 to 60% of the corresponding body width, *pars distalis ca.* 17 µm long, *pars proximalis vaginae* about as high as its width (16 to 18 µm), and a transverse slit in the vulva. Tail conical, dorsally convex, with wide-rounded end.

#### Males

Rare, only one out of 19 females. Similar to females in general morphology, except for the reproductive system and posterior body end being strongly curved ventrally. The reproductive system is composed of two opposed testes. Spicules 71 µm long, 13 µm wide or ca. 5.5 times longer than wide. Supplements composed of a cloacal pair at 12 µm distance from the cloacal opening and 12 single ventral supplements ending at 20 µm distance from the cloacal pair. Tail conical, dorsally convex, ventrally concave, with a wide-rounded tip.

#### Juveniles

**Figure 3:**
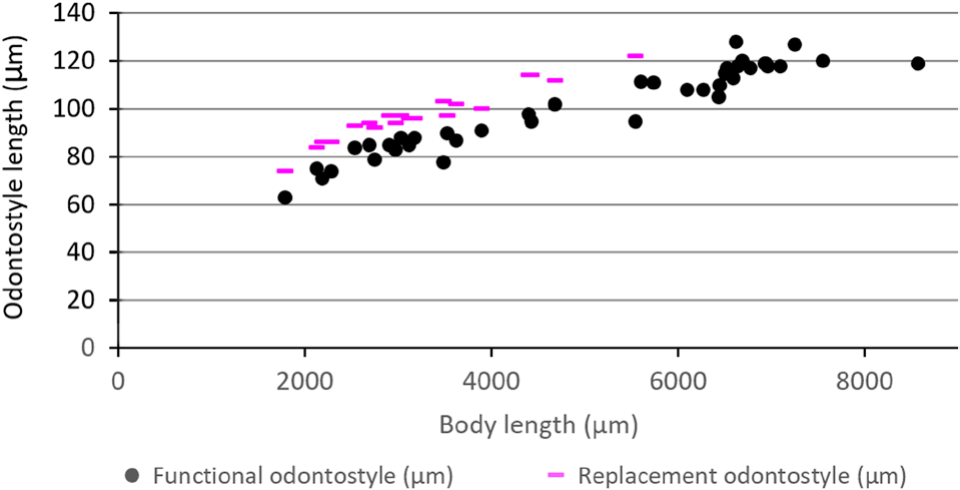
Correlation of functional and replacement odontostyle to body length in juveniles and females of Iranian population of *Longidorus proximus* (Sturhan and Argo, 1983). In each stage the length of replacement odontostyle is equal to the length of functional odontostyle in the next stage.

All four juvenile developmental stages were recovered and identified. The diagram of the correlation of body size and functional and replacement odontostyle of the juveniles and females is given in Figure 3. Their general morphology looks similar to that of females, except for a smaller body size, having the replacement odontostyle and underdeveloped reproductive system. The first juvenile developmental stage (J1) is characterized by having a replacement odontostyle lying on the odontophore, with its tip close to the base of the functional odontostyle. In the other juvenile developmental stages (J2–J4), the tip of the replacement odontostyle is located farther from the base of the functional odontostyle. The tail is conical in all stages. J1 has a narrower tail compared to the tail in other three stages, with a dorsally convex and ventrally concave outline and rounded tip. The tail in J2–J3 is conical, dorsally convex and ventrally slightly convex, with a wide-rounded end.

### Molecular characterization and phylogenetic position of the Iranian population of *Longidorus proximus*

**Figure 4:**
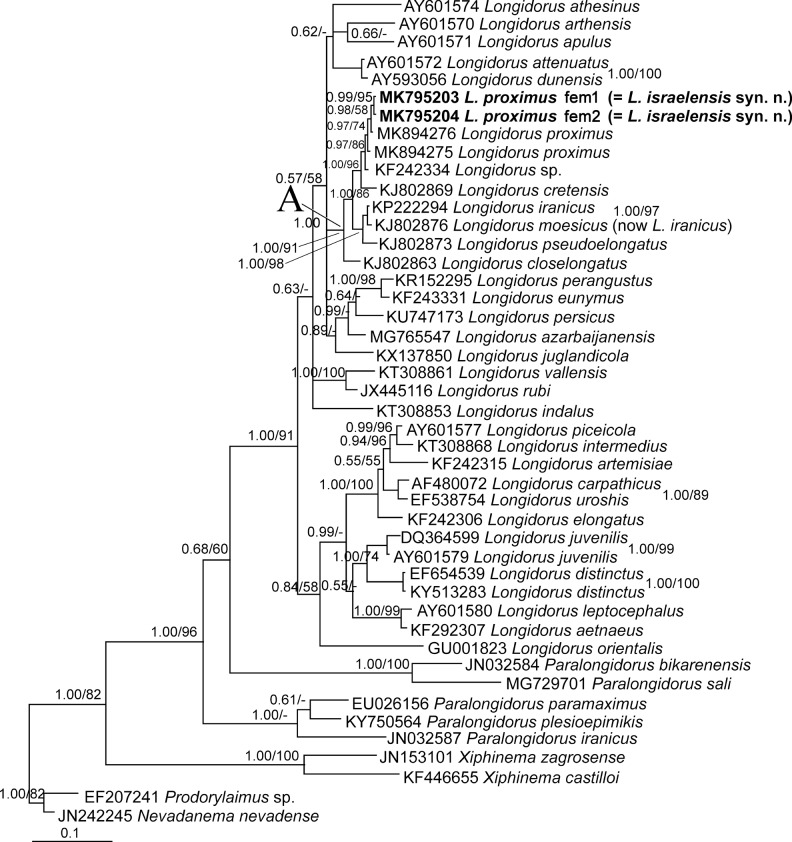
Bayesian tree inferred under the GTR+G+I model from LSU rDNA D2-D3 expansion domains of Iranian population of *Longidorus proximus* (Sturhan and Argo, 1983). Posterior probability and bootstrap values exceeding 50% are given on appropriate clades in the form BPP/BS. The new sequences are in bold font.

Sequencing of LSU rDNA D2-D3 expansion domains of two females of *L. proximus* yielded two single fragments, 701 (fem1, MK795203) and 757 (fem2, MK795204) nt long. The longer size of the latter sequences was due to the longer read of the corresponding PCR product while sequencing. Both sequences were identical in their overlapping region after aligning. Sequencing of the ITS1 rDNA fragment of a female yielded a single fragment, 623 nt long (MK795202). A BLAST search using one of the newly obtained D2–D3 sequences (fem2, MK795204) revealed it had 99.74 to 99.84% identity with the unpublished isolates of *L. proximus* (MK894274-MK894276) and that, in comparison with them, it had at maximum one indel. The other highly identical sequences belonged to *Longidorus* sp. (KF242334) with 99.46% identity and *L. cretensis* (Tzortzakakis et al., 2001) (KJ802867) with 98% identity. The BLAST search using the ITS1 sequence revealed it had the maximum (95.5%) identity with *L. sturhani* (Rubtsova et al., 2001) (FJ009680). A total of 53 sequences of *Longidorus* spp., 11 sequences of *Paralongidorus* spp., and two sequences of *Xiphinema* spp. were used for resolving the LSU phylogeny (the reconstructed tree was not shown). A pruned tree based on this tree was reconstructed using 36 sequences of *Longidorus* spp., five sequences of *Paralongidorus* spp., and two sequences of *Xiphinema* spp. as outgroups. This pruned dataset had 821 characters. The phylogenetic tree reconstructed using this dataset is shown in Figure 4. The two newly generated (MK795203, MK795204) and two available sequences (MK894275, MK89427) of *L. proximus* fell in the clade A, including *L. iranicus* (Sturhan and Barooti, 1983), *L. pseudoelongatus* (Altherr, 1976), *L. cretensis*, and *L. closelongatus* Stoyanov, 1964. *Longidorus* sp. (KF242334) was the closest relative to *L. proximus* in this tree and had four indels in comparison with its sequences.

**Figure 5:**
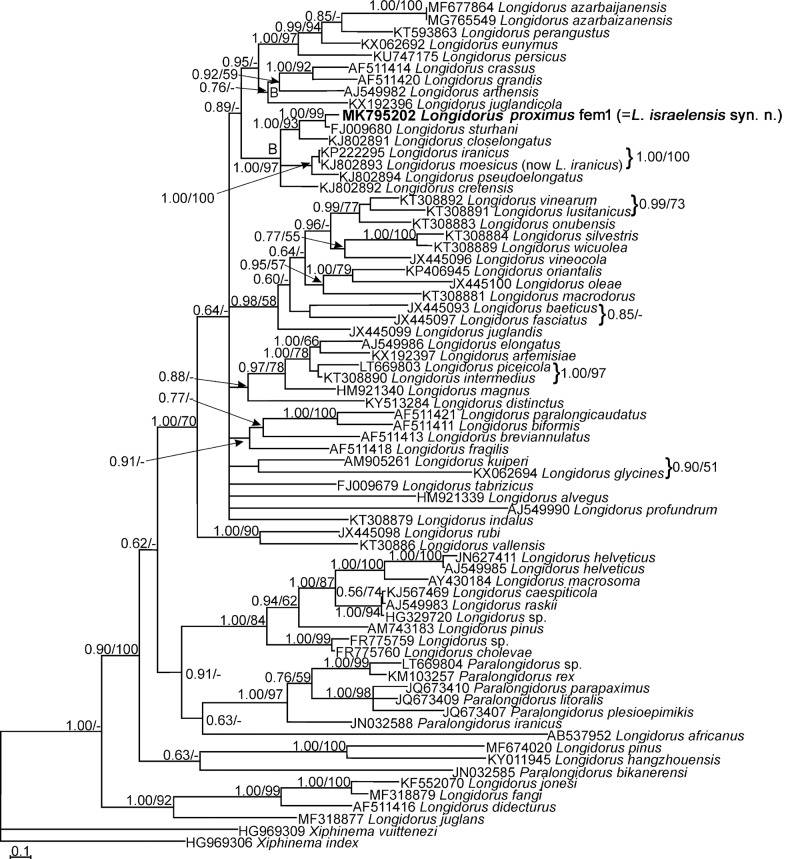
Bayesian tree inferred under the GTR+G+I model from ITS1 rDNA partial sequences of Iranian population of *Longidorus proximus* (Sturhan and Argo, 1983). Posterior probability and bootstrap values exceeding 50% are given on appropriate clades in the form BPP/BS. The new sequence is in bold font.

A total of 62 sequences of *Longidorus* spp., seven sequences of *Paralongidorus* spp., and two sequences of *Xiphinema* spp. as outgroup taxa were used for reconstructing the ITS tree. The corresponding dataset was composed of 1600 characters. Figure 5 represents the phylogenetic tree reconstructed using this dataset. In this tree, *L. israelensis*, *L. sturhani*, *L. pseudoelongatus*, *L. closelongatus*, and *L. iranicus* have formed a highly supported clade (clade B, 1.00/97), and *L. sturhani* is the closest relative to *L. israelensis* based on this fragment.

## Discussion

Currently around 170 nominal species have been described under the genus *Longidorus* (Gharibzadeh et al., 2018). The number of species is still increasing (Archidona-Yuste et al., 2019; Lazarova et al., 2019). The abnormal status of the pharyngeal glands nuclei, i.e. having a larger dorsal gland nucleus located at about the mid-pharyngeal bulb length, and the two smaller ventrosublateral nuclei at posterior position in the pharyngeal bulb, has been reported for few species (Chen et al., 1997). According to Tzortzakakis et al. (2001), this status is seen in *L. closelongatus*, *L. cohni* (Heyns, 1969), *L. iranicus*, *L. proximus*, *L. israelensis* and *L. cretensis*. The two species *L. proximus* and *L. israelensis* have a very close morphology and morphometric data ranges. *L. israelensis* is differentiated from *L. proximus* mainly by its longer odontostyle (125–135 vs 102–112 µm), while with regard to the total stylet range of females and males of the Greece population of *L. proximus* as described by Roca (1986) (103–129 µm), the lengths of the odontostyle of both species are similar. A wide range of odontostyle length of *Longidorus* spp. is already documented (e.g. *L. macrodorus* (Archidona-Yuste et al., 2016), *L. cholevae* (Peneva et al., 2013), *L. perangustus* (Roshan-Bakhsh et al., 2016)). The lengths of the odontophore of J4 and females of both species have been reported to be different (37–46 µm in J4 of *L. proximus* vs 72.5–78.0 µm in J4 of *L. israelensis*, 41–51 µm in females of *L. proximus* vs 67–82 µm in females of *L. israelensis*). The females of the Greece population of *L. proximus* on the other hand have an odontophore length of 54–71 µm, which is more close to the range given for *L. israelensis* and the present Iranian population of *L. proximus* (58–64 µm). Owing to the difficulties involved in reliable measurement of the odontophore in *Longidorus* spp. and *Paralongidorus* spp., it is proposed that delimiting of the species of both genera should not be relied upon by solely basing on this trait. The odontophore length of the females of our population fit well with the length given for the Greece population of *L. proximus* by Roca. With regard to the shared morphology of both species, *L. proximus* and *L. israelensis*, the latter species was regarded as the junior synonym of the former.

The LSU D2-D3 sequences of our *L. proximus* isolates are almost identical to those of other *L. proximus* isolates available in GenBank (at maximum one indel was observed), corroborating the identity of the present population.

The characters of glands’ nuclei in *L. pseudoelongatis,* a member of the clade A in our LSU tree, including *L. closelongatus* group species *sensu* (Tzortzakakis *et al*. (2001)) are not given in its original description. *L. sturhani,* the closest relative to *L. proximus* in the ITS tree, has a normal size and arrangement of the nuclei. Based on our phylogenetic studies, species having a unique arrangement and size of pharyngeal gland nuclei, as already described for the *L. closelongatus* group, are regarded as phylogenetically related (molecular data are currently not available for *L. cohni*).

The polytomous codes of the Iranian population of *Longidorus proximus* according to Chen et al. (1997) are A45, B34, C234, D3, E1, F34, G23, H12, and I2. Compared to the original description of *L. proximus*, the population studied here had no remarkable morphological differences, and the status of odontophore was already discussed. *L. proximus* is morphologically close to *L. paraelongatus* (Altherr, 1974) and both species share similar polytomous identification codes (Chen et al., 1997). The latter species is described based on only one female. Our contact with the Agroscope center in Switzerland to get access to the type material of this species for detailed studies revealed that no type material of the species is available, and because of lacking sufficient morphological data such as the characters of the juveniles, males, pharyngeal gland nuclei, and type material, this species is considered not to be valid.

## Conclusion

In this study, morphological and molecular data were provided for an Iranian population of *L. proximus*, and, based on the morphological and morphometric data, *L israelensis* was proposed as a junior synonym of *L. proximus*. In our phylogenetic analyses using two genomic markers, the species having a unique arrangement and size of pharyngeal gland nuclei showed close phylogenetic affinity too.
